# Targeting the Anti-Apoptotic Protein c-FLIP for Cancer Therapy

**DOI:** 10.3390/cancers3021639

**Published:** 2011-03-29

**Authors:** Ahmad R. Safa, Karen E. Pollok

**Affiliations:** 1 Department of Pharmacology and Toxicology, Indiana University School of Medicine, 980 W. Walnut Street, R3-C524, Indianapolis, IN 46202, USA; 2 Indiana University Simon Cancer Center, Indiana University School of Medicine, 980 W. Walnut Street, R3-C524, Indianapolis, IN 46202, USA; 3 Herman B. Wells Center for Pediatric Research, 980 W. Walnut Street, R3-C524, Indianapolis, IN 46202, USA; E-Mail: kpollok@iupui.edu (K.E.P)

**Keywords:** c-FLIP, apoptosis, death receptors, cancer, chemotherapy

## Abstract

Cellular FLICE (FADD-like IL-1beta-converting enzyme)-inhibitory protein (c-FLIP) is a major resistance factor and critical anti-apoptotic regulator that inhibits tumor necrosis factor-alpha (TNF-alpha), Fas-L, and TNF-related apoptosis-inducing ligand (TRAIL)-induced apoptosis as well as chemotherapy-triggered apoptosis in malignant cells. c-FLIP is expressed as long (c-FLIP_L_), short (c-FLIP_S_), and c-FLIP_R_ splice variants in human cells. c-FLIP binds to FADD and/or caspase-8 or -10 in a ligand-dependent and-independent fashion, which in turn prevents death-inducing signaling complex (DISC) formation and subsequent activation of the caspase cascade. Moreover, c-FLIP_L_ and c-FLIP_S_ are known to have multifunctional roles in various signaling pathways, as well as activating and/or upregulating several cytoprotective signaling molecules. Upregulation of c-FLIP has been found in various tumor types, and its downregulation has been shown to restore apoptosis triggered by cytokines and various chemotherapeutic agents. Hence, c-FLIP is an important target for cancer therapy. For example, small interfering RNAs (siRNAs) that specifically knockdown the expression of c-FLIP_L_ in diverse human cancer cell lines augmented TRAIL-induced DISC recruitment and increased the efficacy of chemotherapeutic agents, thereby enhancing effector caspase stimulation and apoptosis. Moreover, small molecules causing degradation of c-FLIP as well as decreasing mRNA and protein levels of c-FLIP_L_ and c-FLIP_S_ splice variants have been found, and efforts are underway to develop other c-FLIP-targeted cancer therapies. This review focuses on (1) the functional role of c-FLIP splice variants in preventing apoptosis and inducing cytokine and drug resistance; (2) the molecular mechanisms that regulate c-FLIP expression; and (3) strategies to inhibit c-FLIP expression and function.

## 1. Introduction

Cytotoxic anticancer agents continue to serve as the mainstay modality of systemic therapy in treating human malignancies that have disseminated from the primary tumor site and cannot be managed solely by surgical removal or radiation. The major limitation of cancer chemotherapy has proven to be drug resistance, whether acquired by the malignancy after transient disease remission (e.g., after breast cancer chemotherapy), or intrinsic to the malignancy (e.g., colon cancer, pancreatic cancer, glioblastoma, and prostate cancer are typically refractory to cancer chemotherapy). Several mechanisms have been found to cause chemotherapeutic resistance in cancer cells *in vitro* [1-4], but whether these mechanisms are also operative in the same manner *in vivo* requires further study. Understanding the mechanisms of resistance to chemotherapeutic agents will assist in the design of more effective strategies to overcome resistance in cancer cells.

Defects in apoptotic signaling and redundant survival mechanisms in malignant cells contribute to drug resistance in various cancer types [5,6]. Therefore, strategies to lower the threshold for triggering apoptosis in various cancers may lead to new and more effective therapeutic regimens. Acutely induced chemosensitization occurs when a pro-apoptotic signaling program induced in neoplastic cells by a chemotherapy drug includes disabling of a cytoprotective anti-apoptotic response. This is illustrated by our discovery that acute exposure of human leukemia cells to Taxol induced a pro-apoptotic program that entails coordinate caspase activation and downregulation of the anti-apoptotic protein cellular FLICE-like inhibitory protein (c-FLIP), a catalytically inactive caspase-8/-10 homologue [7]. c-FLIP variants are involved in tumor necrosis factor-related apoptosis-inducing ligand (TRAIL) and chemotherapeutic drug resistance in a wide range of human malignancies [7-13]. The fact that Taxol has the added benefit of disabling a specific cytoprotective signal in neoplastic cells in conjunction with inducing apoptosis signaling is consistent with its often superior efficacy compared with other apoptosis-inducing chemotherapy drugs in managing diverse neoplastic diseases. Furthermore, a combination of Taxol/c-FLIP targeted therapy may improve the therapeutic response to Taxol by enhancing downregulation of c-FLIP variants in concert with drug-induced apoptosis signaling [7].

We have reported that upregulation of the pro-apoptotic TRAIL receptor DR5 may actually occur during the development of chemotherapy-induced drug resistance phenotype in cancer cells [14]. Moreover, upregulation of the pro-apoptotic signaling proteins or suppression of specific anti-survival signaling pathways by agents directed to increase pro-apoptotic proteins may acutely induce chemosensitization of resistant cancer cells. For instance, we previously demonstrated TRAIL treatment selectively triggered apoptosis in P-glycoprotein (P-gp ABCB1)-overexpressing multidrug resistant (MDR) cells [14-16]. Moreover, hypersensitivity to TRAIL was either due to (1) increased TRAIL binding to the TRAIL receptor DR5 in these cells compared to their drug sensitive counterparts [15]; or (2) up-regulation of DR5 and concomitant degradation of P-gp [14], the release of cytochrome *c* from mitochondria, activation of caspases-9 and -3 [14], as well as down-regulation of c-FLIP and the DNA-dependent protein kinase catalytic subunit (DNA-PKcs) by activation of caspase-3 [17]. These data also provided important determinants of TRAIL-induced sensitization of MDR cells to MDR-related agents [14,17]. Therefore, these results hold significant clinical implications for the use of TRAIL or TRAIL and chemotherapeutic drugs for treating cancers with the MDR phenotype. TRAIL holds enormous promise as a cancer therapeutic due to its highly selective apoptosis-inducing action on neoplastic *versus* normal cells [18,19]. Moreover, a recently published Phase I clinical trial revealed that recombinant TRAIL administration is safe and well tolerated, and that dose escalation achieved peak TRAIL serum concentrations equivalent to those associated with preclinical antitumor efficacy [20] However, to exploit the opportunity to successfully treat cancers with TRAIL, the problems of TRAIL resistance in a variety of tumor cells must first be overcome [21-23].

It is now recognized that the mechanism of action of chemotherapy drugs often involves the induction of cancer cell apoptosis, and that apoptosis resistance is a major contributing factor in chemotherapeutic drug resistance. Therefore, restoring apoptosis signaling in cancer cells with targeted therapeutics has enormous potential to improve the outcome of cancer chemotherapy by reversing a major mechanism of drug resistance. As we previously reported [24], c-FLIP is a critical target for therapeutic intervention aimed at inhibiting its transcription and posttranscription. In this review, we assess the outlook for improving the outcome of cancer therapy by targeting c-FLIP and exploring the possibility of increasing its degradation and/or decreasing its expression in order to provide a potentially safe approach to treat cancer. Novel modalities of cancer therapy that improve the efficacy of TRAIL as well as chemotherapeutic drugs and lessen the toxicity of these agents by targeting specific c-FLIP isoforms is discussed.

## 2. Apoptosis Signaling Pathways

**Figure 1.**
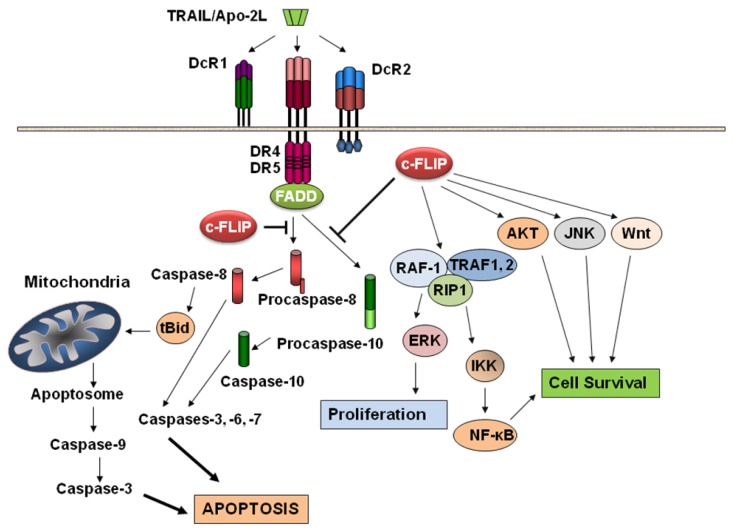
Schematic overview of the multifunctional roles of c-FLIP in the TRAIL-triggered apoptosis pathway as well as activating various anti-apoptotic and cell survival signaling pathways.

Apoptosis is a mechanism of programmed cell death involving signal transduction pathways that induce cells to self-destruct in response to organismal cues, e.g., digit formation in vertebrate limbs during embryonic development, environmental hazards (e.g., radiation-induced DNA damage), or anticancer therapeutics (e.g., chemotherapeutic agents and cytokines). Two well-studied pathways are involved in apoptosis: the mitochondrion-initiated pathway (Figure 1) and the cell surface death receptors pathway (Figure 1) [25-27]. In the mitochondrial pathway, cytochrome *c*, certain caspases, apoptosis-inducing factor, Smac/DIABLO, and other apoptosis-inducing factors are released from the mitochondrial intramembrane space to the cytosol [28]. Once released, cytochrome *c* and dATP bind to apoptotic proteinase-activating factor-1 (Apaf-1), and this complex along with adenine nucleotides promote procaspase-9 autoactivation [29], which in turn activates caspases-2, -3, -6, -7, -8, and -10. Apoptosis triggered by various stimuli requires direct activation of Bax and BAK at the mitochondria by a member of the Bcl-2 homology domain-3 (BH3)-only family of proteins including Bid, Bim, or PUMA [30]. The various anti- and pro-apoptotic members of the Bcl-2 family form a network of interactions that ultimately regulate the permeabilization of the mitochondrial outer membrane and release of apoptogenic factors such as cytochrome *c* to the cytoplasm [31]. Apoptosis initiated by the endoplasmic reticulum (ER) stress signaling pathway is also mainly dependent on the release of cytochrome *c* from the mitochondrial intermembrane space into the cytosol [32]. This release is associated with the opening of the permeability transition pore (PTP) and a collapse in the mitochondrial transmembrane potential (ΔΨ_m_) due to the intake of Ca^2+^ following its release into the cytosol from the ER. Recent work has demonstrated that certain members of the Bcl-2 family are present on the ER where they appear to have a comprehensive function in the maintenance of ER homeostasis, participation in ER stress signal transduction pathways, and apoptosis [32].

In the death receptor-mediated apoptosis pathway (e.g., Fas/Fas ligand interaction or TRAIL/DR5 interaction and cell death), the initiator caspases-8 and -10 activate the downstream caspases including caspase-3 [18,19,24]. Active caspases-8 and -10 are known to cleave a pro-apoptotic Bcl-2 family member, Bid, and the truncated Bid induces mitochondrial cytochrome c release [26-29], thereby linking the two pathways. After activation, both caspases-8 and -9 activate caspase-3, which in turn cleaves other caspases and many cellular proteins including fodrin, various kinases, poly(ADP-ribose) polymerase, gelsolin, and DNA fragmentation factor-45 (DFF45) [26,33-37]. A third pathway has also been identified [38]. In this pathway, as Slee *et al.* [38] showed, BID is cleaved in response to several death-inducing stimuli (staurosporine, UV radiation, cycloheximide, etoposide) and that BID cleavage was blocked by Bcl-2, suggesting that degradation of BID occurred distal to cytochrome c release. Moreover, addition of cytochrome c to Jurkat post-nuclear extracts triggered cleavage of BID at Asp-59 which was catalysed by caspase-3 rather than caspase-8. These results provide evidence that caspase-3 mediated cleavage of BID represents a feedback loop for the amplification of mitochondrial cytochrome c release during cytotoxic drug and UV radiation-induced apoptosis [38].

## 3. Cellular FLICE-Like Inhibitory Protein (c-FLIP)

### 3.1. Structure of c-FLIP

**Figure 2.**
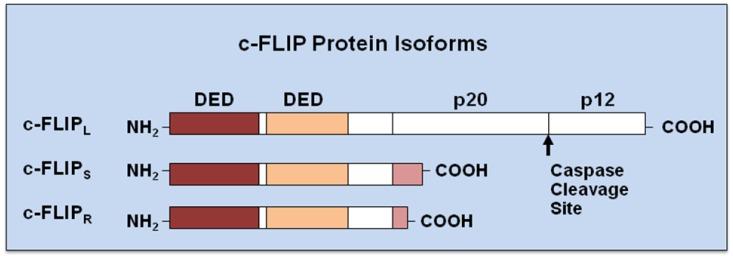
Structures of c-FLIP isoforms. Three c-FLIP isoforms, c-FLIP_L_, c-FLIP_s_, and c-FLIP_R_, contain two death effector domains (DEDs) at their N-termini. In addition to two DEDs, c-FLIP_L_ contains a large (p20) and a small (p12) caspase-like domain without catalytic activity. c-FLIP_S_ and c-FLIP_R_ consist of two DEDs and a small C-terminus [36].

Originally, viral FLICE-inhibitory proteins (v-FLIPs) were identified by a bioinformatic search for novel virus-encoded apoptotic regulatory molecules containing a death effector domain (DED) [39-42]. Following the characterization of v-FLIPs, the mammalian cellular homologue was identified and called c-FLIP [43]. c-FLIP, also known as Casper, iFLICE, FLAME-1, CASH, CLARP, MRIT or usurpin [44,45], has 13 distinct splice variants [44], three of which are expressed as proteins: the 26 kDa short form (c-FLIP_S_), the 24 kDa form of c-FLIP (c-FLIP_R_), and the 55 kDa c-FLIP_L_ [24,44,45] (Figure 2). The structures of c-FLIP_S_ and the v-FLIP proteins are similar, except that the two DEDs of c-FLIP_S_ are followed by 20 amino acids that appear to be crucial for its ubiquitaation and targeting for proteasomal degradation [46]. c-FLIP_R_ also contains two DEDs but lacks the additional carboxy (C)-terminal amino acids that are present in c-FLIP_S_. The C-terminus of c-FLIP_L_ is longer than that of c-FLIP_S_ and closely resembles the structure of caspases-8 and -10 [47,48], but this region of c-FLIP_L_ does not contain a functional caspase domain. This lack of caspase activity is the result of several amino acids substitutions, particularly the crucial cysteine residue in the catalytic domain which is necessary for the catalytic activity of caspases [47]. Additionally, c-FLIP_L_ harbors a caspase-8 cleavage site at position Asp-376 (LEVD); c-FLIP_L_ cleavage at this site produces the proteolytic fragment variant p43c-FLIP [48,49]. The C-terminal region of c-FLIP_S_ and c-FLIP_R_ play a crucial role in ubiquitnation and degradation as well as the anti-apoptotic function of these isoforms [50,51]. All three isoforms of c-FLIP can be recruited to the DISC through an interaction of their tandem DED domains with the adaptor protein FADD. Recently, Ueffing *et al.* [52] reported a functional single nucleotide polymorphism (SNP) in the human c-FLIP gene (rs10190751 A/G), located in the 3′ splice consensus of intron 6, which determines c-FLIP_S_ production. Analysis of the rs101900751 G/A variation in follicular lymphoma patients indicates that rs10190751 A, which directs expression of the c-FLIP_R_ isoform, is associated with increased risk for this disease [52].

### 3.2. Transcription and Translation of c-FLIP

Transcriptional activation of c-FLIP can be mediated by various signal transducers, including TNF ligands, growth factors, interleukins, chemokines, and chemotherapeutic agents [24]. Several transcription factors are known to transcriptionally regulate the *c-FLIP* gene [24,45]. These include NF-κB, p53 tumor suppressor protein, p63, E2F1, c-myc, IRF5, c-Fos, nuclear factor of activated T cells (NFAT), heterogeneous nuclear ribonucleoprotein K (hnRNP k), the forkhead transcription factor FOXO3a [42], Early growth response-1(EGR1), androgen receptor (AR), E2F, AP-1, and SP1 [24,45]. While NF-kB, p63, NFATc2, EGR1, hnRNP K, AR and SP1 are known to induce c-FLIP expression, c-myc, Foxo3a, c-Fos, IRF5, and SP3 suppress c-FLIP transcription [24,45]. p53 may transcriptionally upregulate the c-FLIP gene and also promote the degradation of c-FLIP protein [53,54]. Intriguingly, c-FLIP_S_ was highly induced upon activation of T cells, primarily via the calcineurin-NFAT pathway [55]. Moreover, the human T-cell leukemia virus type 1 (HTLV-1)Tax protein up-regulating c-FLIP expression in HTLV-1-infected cells through activation of NF-κB [56].

Li *et al.* [57] reported that c-FLIP_L_ is transcriptionally regulated by the activator protein-1 (AP-1) family member protein c-Fos, and that MG-132, an inhibitor of the proteasome, sensitizes TRAIL-resistant prostate cancer cells by inducing c-Fos and repressing c-FLIP_L_. Moreover, c-Fos, which is activated by MG-132, negatively regulates *c-FLIP_L_* by direct binding to the putative promoter region of the c-FLIP_L_ gene. In addition to activating c-Fos, MG-132 also activates c-Jun, another AP-1 family member. c-Fos heterodimerizes with c-Jun to form an AP-1 complex that represses transcription of c-FLIP_L_.

E2F1, a transcription factor that plays a crucial role during S phase progression and apoptosis, triggers apoptosis in various lung adenocarcinoma cell lines by specifically downregulating of c-FLIP_S_ leading to caspase-8 activation at the DISC [58]. Moreover, the SC35 splicing factor, which is a direct transcriptional target of E2F1, is involved in downregulation of c-FLIP_S_ [59]. The specific overexpression of c-FLIP_S_ is also seen in human lung adenocarcinomas with low levels of E2F1 [58]. Delineating the role of SC35 in regulating the expression of c-FLIP_S_ will be very significant, not only for understanding how alternative splicing of the c-FLIP gene occurs, but also to possibly decrease the level of c-FLIP_S_ by modulating SC35 expression.

c-FLIP_S_ is also regulated at the translational level. Panner *et al.* [60,61] showed that TRAIL resistance in glioblastoma multiforme cells (GBM) is the result of c-FLIP_S_ overexpression, and that activation of the Akt mammalian target of rapamycin (mTOR)-p70 S6 kinase 1 (S6K1) pathway leads to increased translation of the c-FLIP_S_ protein. Conversely, inhibition of mTOR or its target S6K1 suppressed polyribosomal accumulation of c-FLIP_S_ mRNA, c-FLIP_S_ protein expression, and promoted TRAIL resistance in GBM cells. An mTOR-independent pathway can also act through a Ral effector protein, RalBP1 to suppress cdc42-mediated activation of S6 kinase and the translation of the c-FLIP_S_ protein [61,62]. Moreover, it has been shown that Rocaglamide (Roc) sensitizes resistant adult T-cell leukemia/lymphoma (ATL) cells to DR4- and DR5-mediated apoptosis by translational suppression of c-FLIP_S_ [63,64] through inactivation of the translation initiation factor 4E (eIF4E) [64].

### 3.3. c-FLIP Degradation

c-FLIP isoforms are short lived proteins whose stability is subject to isoform-specific regulation. c-FLIP is predominately degraded by the ubiquitin-proteasome degradation system [24,45,50]. Both c-FLIP isoforms can be degraded by the proteasome, but c-FLIP_S_ appears to be particularly sensitive to ubiquitination and proteasomal degradation, partly due to two crucial lysine residues in the C-terminal 20 amino acids that are unique to c-FLIP_S_ [50]. The sensitivity of c-FLIP_S_ to ubiquitin-mediated degradation adds a novel concept to DISC regulation and its control of apoptosis [50].

Expression of c-FLIP_L_ and c-FLIP_S_ is also regulated by JNK activation via the E3 ubiquitin ligase Itch [24,45,65] under the control of JNK, polyubiquitinates c-FLIP to target it for degradation at the proteasome [65]. Phosphorylation events also play important roles in the regulation of c-FLIP protein levels. For instance, protein kinase C phosphorylation at the serine 193 (S193) residue of the c-FLIP_S_ isoform inhibits its polyubiquitination, stabilizes c-FLIP_S_ levels, and increases cell survival [66]. S193 phosphorylation was markedly increased by treatment with the PKC activator 12-*O*-tetradecanoylphorbol-13-acetate and decreased by inhibition of PKCα and PKC*β*. Phosphorylation of S193 residue also decreased the ubiquitination of c-FLIP_L_ but did not affect its stability, indicating that S193 phosphorylation has a different function in c-FLIP_L_ than c-FLIP_S_. Moreover, Wang *et al.* [67] showed that pretreatment with the PKCδ-selective inhibitor rottlerin or transfection with PKCδ siRNA inhibited phorbol myristate acetate (PMA)-induced c-FLIP expression, which identifies a role for PKCδ in c-FLIP induction. These authors demonstrated a critical role for PKCδ/NF-κB in the induction of c-FLIP in human colon cancer cells. Downregulation of AMP-activated protein kinase also triggers ubiquitination and proteasome degradation of c-FLIP [68].

### 3.4. Upregulation of c-FLIP in Human Cancers

Increased expression of c-FLIP has been shown in cell lines from various types of cancers including colorectal [69,70], pancreatic [71,72], ovarian [73,74], gastric [75], breast [76,77], prostate [78], melanoma [79], glioblastoma [80], and it is implicated in TRAIL resistance and chemotherapy resistance. Gastric cancer SNU-216 cells [12], some pancreatic cancer cell lines [71], breast cancer cells [76,77], and leukemia cells [7,16] express high levels of c-FLIP_L_ and c-FLIP_S_. FLIP_S_ is also a key suppressor of TRAIL-induced apoptosis in human glioblastoma multiforme (GBM) cell lines and xenografts [60]. Furthermore, elevated levels of c-FLIP in tumor tissue from patients with colorectal cancer [80,81], bladder urothelial cancer [82], cervical cancer [83], Burkitt's lymphoma [84], non-Hodgkin's lymphoma [85], and head and neck squamous cell carcinoma (HNSCC) [86], and have been correlated with a poor clinical outcome and could be a reliable prognostic factor in these type of cancer. Overexpression of c-FLIP is also seen in gastric cancer and plays an important role in lymph node metastasis, which ultimately contributes to the tumor progression [87]. c-FLIP is expressed in pancreatic intraepithelial neoplasm lesions as well as in pancreatic ductal adenocarcinomas, whereas normal pancreatic ducts were consistently negative for c-FLIP expression [71].

### 3.5. c-FLIP Function

#### 3.5.1. c-FLIP prevents apoptosis

Studies with animal models have revealed that c-FLIP plays an important role in T cell proliferation and heart development [88,89]. Moreover, abnormal c-FLIP expression has been found in various diseases such as cancer, multiple sclerosis, Alzheimer's disease, diabetes mellitus, and rheumatoid arthritis [24,44]. c-FLIP is also thought to be the main causal factor of “immune escape” [90]. c-FLIP is involved in TRAIL, Fas, TNF-α, and chemotherapeutic drug resistance in a wide range of human malignancies [24,44,72,91]. Moreover, studies using c-FLIP-deficient mice support a dual function for c-FLIP_L_ by confirming a role for c-FLIP in Fas L, TNF-α-induced and apoptosis and revealing that c-FLIP has a similar function to caspase-8 in heart development [89]. Nonetheless, a now extensive literature encompassing diverse types of human cancer cells indicates that the action of c-FLIP is generally anti-apoptotic in cancer cells. Furthermore, interference with c-FLIP expression sensitizes tumor cells to death ligands and chemotherapy in experimental models [24,45,91-94]. In addition to its function as an apoptosis modulator, c-FLIP exerts other cellular functions including increased cell proliferation and tumorigenesis [24,45,48,94-96] (Figure 1).

While the precise mechanism of c-FLIP regulation of apoptosis remains elusive, the profound structural differences between human c-FLIP variants clearly indicate distinct regulatory roles for c-FLIP_L_ and c-FLIP_S_ in apoptosis. In fact, c-FLIP_S_ inhibits TRAIL-induced DISC formation and apoptosis [24,45,48], while c-FLIP_L_ is responsible for the above described dual functions whereby it inhibits Fas-induced caspase-8 activation when expressed at high levels, but enhances caspase-8 activation when its expression level is low [24,45]. These opposing c-FLIP_L_ functions may reflect observations that c-FLIP_L_ activates caspases-8 and -10 *in vitro* by forming heterodimeric enzyme molecules with a substrate specificity and catalytic activity indistinguishable from caspase-8 homodimers, despite the fact that c-FLIP_L_ is protease dead [24,97,98]. Recent reports have clearly demonstrated that c-FLIP_S_ also plays a central role in preventing cancer cell apoptosis. c-FLIP_S_ has been shown to inhibit oxaliplatin-induced apoptosis through the sustained XIAP protein level and Akt activation [99]. c-FLIP_S_ also suppresses apoptosis by inhibiting caspase-8 activation [100-102], although at different levels of procaspase-8 process [103,104]. c-FLIP_L_ induces a conformation of procaspase-8 that triggers partial but incomplete proteolytic processing, while in contrast, c-FLIP_S_ even prevents partial procaspase-8 activation at the DISC [8]. Using an *in vitro* induced proximity assay, Boatright *et al.* [97] provide evidence that c-FLIP_L_ is an activator of caspase-8/-10 and demonstrate that the resulting heterodimer is enzymatically active with a substrate specificity identical to that of the caspase-8 homodimer.

We recently discovered that c-FLIP_L_ interacts with DR5, FADD, and caspase-8 forming an apoptotic inhibitory complex (AIC) in MCF-7 breast cancer cells [76]. Moreover, silencing the c-FLIP gene by a specific siRNA leads to death ligand-independent but DR5-, FADD-, and caspase-8- and -9-dependent apoptosis in these cells. Furthermore, we showed that the knockdown of c-FLIP expression inhibits breast cancer cell proliferation and triggers spontaneous apoptosis by activating both the death receptor and mitochondrial pathways [76]. Our data support the previous report by Jin *et al.* [105] demonstrating that the peptide corresponding to the DR5 binding domain of c-FLIP_L_ induces apoptosis in cancer cells. Therefore, inhibiting the interaction of DR5 and c-FLIP_L_ by peptides or small molecule inhibitors should provide a mechanism by which tumor selective apoptosis can be achieved. Interestingly, a recent report also identified a checkpoint of the autophagy pathway where cellular and viral FLIPs could limit the Atg3-mediated step of LC3 ubiquitin-like protein conjugation to regulate autophagosome biogenesis. Furthermore, the c-FLIP-derived short peptides hold promise as new cancer therapeutic agents since they induced growth inhibition by binding to and effectively suppressing Atg3-c-FLIP interactions [106].

#### 3.5.2. c-FLIP augments cytoprotective pathways

As shown in Figure 1, c-FLIP activates several cytoprotective signaling pathways involved in regulating cell survival, proliferation, and carcinogenesis. Overexpression of c-FLIP_L_ activates NF-κB and ERK signaling by binding to adaptor proteins in each pathway, such as TNFR-associated factors 1 (TRAF1) and 2 (TRAF2), receptor-interacting protein 1 (RIP), and Raf-1 [48,107] (Figure 1). The caspase-8 processed N-terminal fragment of c-FLIP_L_ (p43cFLIP) is more efficient than c-FLIP_L_ at recruiting TRAF2 and RIP1, leading to more robust NF-κB activation [48,49,108,109]. Golks *et al.* [110] showed that in nonapoptotic cells, c-FLIP and the procaspase-8 heterodimer result in a novel NH_2_-terminal fragment of c-FLIP (p22-FLIP) which is the key mediator of NF-κB activation by binding directly to the IKK complex. These results provide a new mechanism of c-FLIP-mediated NF-κB activation. Recently, Chang *et al.* [111] demonstrated that TNF-α-mediated JNK activation increases turnover of the NF-κB-induced c-FLIP. This is not the result of direct c-FLIP phosphorylation, but rather depends on JNK-mediated phosphorylation and activation of the E3 ubiquitin ligase Itch which specifically ubiquitinates c-FLIP and induces its proteasomal degradation. Thus, JNK antagonizes NF-κB during TNF-α signaling by promoting the proteasomal elimination of c-FLIP_L_.

Akt is a serine-threonine kinase that plays a major role in transducing cellular survival signals and also regulates a number of proteins involved in the apoptotic signaling pathways. Recent results showed that Akt interacts with c-FLIP_L_ protein and that c-FLIP_L_ enhances anti-apoptotic Akt functions by modulating Gsk3β activity. Moreover, through its effects on Gsk3β, c-FLIP_L_ overexpression in cancer cells induced resistance to TRAIL. This effect is mediated by regulation of p27(Kip1) and caspase-3 expression [112]. Downregulation of the DNA-PK/Akt pathway was also reported to correlate with high responsiveness to TRAIL-mediated growth inhibition and apoptosis [113]. siRNA-mediated suppression of DNA-PKcs or treatment with 4,5-dimethoxy-2-nitrobenzaldehyde (DMNB), a specific inhibitor of DNA-PK, led to decreased phosphorylation of Akt and Bad (a target molecule of Akt), increased expression of DR4/DR5, and down-regulation of c-FLIP [113]. Therefore, inhibition of the DNA-PK/Akt pathway may have clinical usefulness in treating TRAIL-resistant cancer cells. [97].

**Figure 3.**
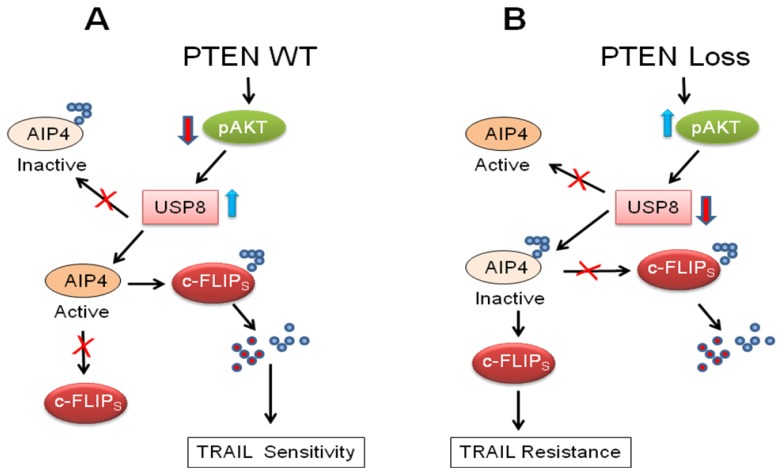
Schematic model of PTEN-mediated control of c-FLIP_S_ ubiquitination and TRAIL sensitivity. (**A**). USP8 interacts with AIP4 which can ubiquitinate c-FLIP_S_ leading to its degradation; (**B**). Increase in pAkt decreases USP8 expression, turns off the USP8/AIP4 ubiquitin switch, resulting in c-FLIP_S_ accumulation. Modified from Panner *et al.* [114].

Panner *et al.* [114] initially reported that a novel phosphatase and tensin homologue (PTEN)-Akt-atrophin-interacting protein 4 (AIP4) pathway regulates c-FLIP_S_ ubiquitination and stability in glioblastoma multiforme (GBM) cell lines and xenografts. However, how PTEN and Akt are linked to AIP4 activity was unclear. Recently, these authors described a second regulator of ubiquitin metabolism, the ubiquitin-specific protease 8 (USP8) which is a downstream target of Akt, and it links Akt to AIP4 and the regulation of c-FLIP_S_ stability [115] (Figure 3). Overexpression of USP8 increased c-FLIP_S_ ubiquitination, decreased FLIP_S_ half-life, decreased FLIP_S_ steady-state levels, and decreased TRAIL resistance (Figure 3). Therefore, PTEN appears to use control of ubiquitination to regulate TRAIL sensitivity in GBM cells.

c-FLIP_L_ also interacts with Daxx (a death domain-associated protein that has been implicated in proapoptosis and transcriptional regulation) and prevents Fas-induced JNK activation [116]. Thus, c-FLIP_L_ acting on both the FADD- and Daxx-mediated signaling pathways may be involved in completely inhibiting Fas-induced cell death. Furthermore, Nakajima *et al.* [117] demonstrated that c-FLIP_L_ directly interacts with a JNK activator, MAP kinase kinase 7 (MKK7), in a TNF-α-dependent manner and inhibits the interactions of MKK7 with MAP/ERK kinase kinase 1 (MEKK1), apoptosis-signal-regulating kinase 1, (ASK1) and TGF-β-activated kinase 1. This interaction of c-FLIP_L_ with MKK7 might selectively suppress JNK activation (Figure 1).

Another regulator of the c-FLIP expression is the calcium/calmodulin-dependent protein kinase II (CaMK II) which mediates the upregulation of c-FLIP, thereby protecting cancer cells from TRAIL-induced apoptosis. Treating resistant cells with the CaMK II inhibitor KN-93 inhibited CaMK II activity, reduced c-FLIP expression, inhibited c-FLIP phosphorylation, and rescued Fas agonistic antibody (CH-11) sensitivity [118,119]. Targeting this pathway may provide novel therapeutic strategies in treating cancers with upregulated CaMK II. Interestingly, phosphorylation of c-FLIP variants by CaMK II appears to promote c-FLIP_L_ recruitment to the DISC and inhibit TRAIL-induced apoptosi [118,119], but phosphorylation of c-FLIP_L_ by protein kinase C or the bile acid glycochenodeoxycholate results in decreased c-FLIP_L_ recruitment to the DISC and increased the sensitivity of hepatocellular carcinoma cells to TRAIL-triggered apoptosis [120]. Thus, the particular site of phosphorylation on c-FLIP_L_ appears to influence the functional outcome of this protein on apoptosis.

Increased expression of c-FLIP can alter cell cycle progression and enhance cell proliferation and carcinogenesis [121,122] (Figure 1). Overexpression of c-FLIP_L_ inhibited the ubiquitination and proteasomal degradation of β-catenin, resulting in an increase in the target gene cyclin D1, colony formation, and invasive activity in prostate cancer cells. The c-FLIP/β-catenin/cyclin D1 signals contributing to colony formation and invasion were reversed by selective silencing of c-FLIP expression [123]. Similarly, c-FLIP_L_, in cooperation with FADD, enhances canonical Wnt signaling by inhibiting proteasomal degradation of β-catenin, thus suggesting a new mechanism involved with tumorigenesis [123]. Recent results also suggest a role for nuclear c-FLIP_L_ in the modulation of Wnt signaling [124]. Interestingly, a deficiency in the adenomatous polyposis coli (APC) gene and subsequent activation of β- catenin can also lead to repression of c-FLIP expression through activation of c-Myc [125], c-FLIP upregulation may contribute to the carcinogenesis and aggressiveness of endometrial carcinomas and may serve as a useful prognostic factor for this tumor [24,126]. Wang *et al.* [127] demonstrated that c-FLIP overexpression is also significantly related to the presence of high-risk human papillomavirus (HR-HPV) infection during the progression of cervical squamous cell cancer and that c-FLIP is an early marker of cervical carcinogenesis. Moreover, HPV16 E2 protein interacts with and abrogates the apoptosis inhibitory function of c-FLIP and renders cervical cancer cell lines hypersensitive to Fas/FasL apoptosis. Overexpression of c-FLIP rescues cervical cancer cells from apoptosis induced by human HPV16 E2 protein expression [112]. This observation is greatly significant for developing therapeutic strategies to silence c-FLIP for intervention with cervical carcinogenesis [128]. Furthermore, overexpression of c-FLIP_L_ also increases the hypoxia-inducible factor-1α (HIF1α) [129]. Overexpression of HIF1α can result in regulation of genes responsible for global changes in cell proliferation, metastasis, and invasion. Moreover, c-FLIP overexpression accelerated progression to androgen independence by inhibiting apoptosis in LNCaP prostate tumors implanted in nude mice [130].

Accumulating information clearly demonstrates that c-FLIP_S_ plays a major role in causing resistance to death ligands and chemotherapeutic agents. Park *et al.* [131] reported that MEK1/2 inhibitors synergistically interacted with the heat shock protein 90 (HSP90) inhibitor, geldanamycins [17-allylamino-17-demethoxygeldanamycin (17AAG) and 17-dimethylaminoethylamino-17-demethoxy-geldanamycin], to kill hepatoma and pancreatic carcinoma cells. Treatment of cells with MEK1/2 inhibitors and 17AAG reduced expression of c-FLIP_S_ that was connected to loss of MEK1/2 and AKT function. Moreover, overexpression of c-FLIP_S_ or Inhibition of caspase-8 abolished cell killing by MEK1/2 inhibitors and 17AAG. Interestingly, Panner *et al.* [132] reported that HSP90α recruits c-FLIP_S_ to the death-inducing signaling complex (DISC) and contributes to TRAIL resistance. Furthermore, combinations of low doses of sorafenib and vorinostat increased CD95 surface levels and CD95 association with caspase-8 and knockdown of CD95 or FADD expression reduced sorafenib/vorinostat cell death [133]. Signaling by CD95 caused protein kinase R (PKR)-like endoplasmatic reticulum kinase (PERK) activation that was responsible for both promoting caspase-8 association with CD95 and increased eIF2α phosphorylation. Suppression of eIF2α function abolished drug combination lethality. Cell killing was paralleled by PERK-and eIF2α-dependent lowering of c-FLIPs protein levels while overexpression of c-FLIP_S_ maintained cell viability [133]. Similarly, Zhang *et al.* [134] showed that expression of phosphorylation-insensitive eIF2α-S51A blocked sorafenib- and vorinostat-induced suppression of c-FLIP_S_ levels and overexpression of c-FLIP_S_ abolished lethality. Overexpression of c-FLIP_S_ function suppressed cell death by the multinuclear platinum chemotherapeutic BBR3610 [135].

#### 3.5.3. c-FLIP increases cell motility

Another important role of c-FLIP is its involvement in increasing cancer cell motility. The role of c-FLIP in cell motility has been investigated using a c-FLIP-specific siRNA. Shim *et al.* [136] showed that siRNA-mediated down-regulation of c-FLIP_L_ correlated with increased levels of reactive oxygen species (ROS), while over-expression of c-FLIP_L_ triggered the opposite effect. ROS generated by silencing c-FLIP induced phosphorylation of Akt and impaired cell motility [136]. The role of c-FLIP in the motility of HeLa cells was also shown using siRNA directed against c-FLIP. Silencing c-FLIP_L_ but not c-FLIP_S_ inhibited the adhesion and motility of the cells by activating FAK and extracellular regulated kinase (ERK), and increasing MMP-9 expression [137]. Additional evidence demonstrating the role of c-FLIP_L_ in triggering cell motility was recently provided in ovarian tumors [73]. In these tumors, c-FLIP_L_ played a role in chaperoning tumor cells from immunosurveillance and increasing their invasive potential by augmenting cell motility [73].

#### 3.5.4. c-FLIP triggers epithelial-mesenchymal transition (EMT)

EMT is a process that induces morphological and genetic changes of cancer cells from an epithelial to a mesenchymal phenotype, which forms the basis for the metastatic potential of tumor cells [138]. Various tumor microenvironmental factors, including cytokines, growth factors, and chemotherapeutic agents trigger EMT [138], and this process is believed to be partly responsible for the chemotherapy-resistant phenotype [138,139]. A cancer-associated antigen gene (CAGE) which is widely expressed in various cancer tissues and cancer cell lines regulates expression of EMT-related proteins through ERK, Akt and NF-kB [140,141]. Snail, an EMT-related protein, mediates the effect of CAGE by inducing matrix metalloproteinase-2 (MMP-2) and cancer cell motility. Interestingly, c-FLIP mediates the effect of CAGE on the induction of MMP-2 and cell motility by the induction of Snail [141].

### 3.6. c-FLIP as a Therapeutic Target for Cancer Treatment

Ectopic expression of c-FLIP variants decreased apoptosis caused by death ligands and anticancer agents [24], indicating that overexpression of these proteins may cause resistance to multiple anticancer drugs. Therapeutic modalities that lower the threshold of cancer cell apoptosis should lead to more effective cancer treatment. For example, strategies to inhibit the expression of c-FLIP variants not only trigger apoptosis in certain cancer types, but also sensitize cancer cells to chemotherapeutic agents, potentially allowing lower doses to be administered to patients and decreasing drug-induced systemic toxicities. Therefore, c-FLIP variants are critical apoptosis regulators that can serve as targets for small molecule inhibitors that downregulate their expression and serve as effective targeted therapeutics for cancer treatment. In order to support this hypothesis, our *in vivo* results showed that injecting liposomal complexes of c-FLIP-specific siRNA into MCF-7 xenografts eliminated the neoplastic cells without affecting the normal stromal and fibroblastic cells [142]. There does not appear to be a “handle” to inhibit c-FLIP function with small molecule ligands since, as discussed above, c-FLIP has significant structural similarity to caspase-8. This resemblance with caspase-8 makes c-FLIP protein a very difficult target for drugs to inhibit its function, since small molecules capable of blocking c-FLIP's recruitment to the DISC could also inhibit the recruitment of caspase-8, and as a result inhibit apoptosis. Therefore, to reduce or inhibit c-FLIP expression, small molecules which target c-FLIP without inhibiting caspases-8 and -10 are needed.

Small molecule therapeutics that selectively downregulate c-FLIP_S_ or c-FLIP_L_ and gene therapy strategies that knock down a specific c-FLIP variant have been used to downregulate these variants. Developing these innovative therapeutic strategies in conjunction with TRAIL and chemotherapeutic agents could potentially overcome the barrier of dose-limiting toxicity in cancer chemotherapy. TRAIL or chemotherapy resistance in diverse cancer cell types can be reversed by parallel treatment with agents known to downregulate c-FLIP variants. As discussed below and shown in Table 1 and  Table 2, c-FLIP variants can be inhibited by compounds that inhibit their transcription or translation, trigger their degradation, or by c-FLIP-specific small interfering RNA (siRNA) which sensitize a wide range of cancer cell types to TRAIL and chemotherapy-induced apoptosis.

#### 3.6.1. c-FLIP transcriptional regulators for cancer therapy

As shown in Table 1, DNA damaging agents are promising drugs with regard to downregulating levels c-FLIP variants. Pretreatment with chemotherapeutic drugs including cisplatin, doxorubicin, or topoisomerase I inhibitors (camptothecin, 9-NC, irinotecan) downregulated c-FLIP variants expression in various tumor cells by inhibiting its transcription and rendering cells sensitive to death receptor-triggered apoptosis (Table 1) [69,143-148]. Successful inhibition of malignant cell growth and apoptosis induction using histone deacetylase inhibitor (HDACi) compounds has highlighted the potential use of these compounds as anticancer agents. Several HDACi have been shown to downregulate c-FLIP expression in various cancer cells at the transcriptional and translational levels [149-152]. Among these, suberoylanilide hydroxamic acid (SAHA, vorinostat) is the most promising HDACi that causes robust inhibition of c-FLIP variants [149]. Recent results demonstrated that TRAIL-triggered apoptosis in breast cancer cells is blocked at the level of apical activation of caspase-8, and that SAHA enhances the TRAIL-induced processing and activation of procaspase-8. Interestingly, degradation of c-FLIPL and c-FLIPS by an ubiquitin/proteasome-dependent Itch/AIP4-independent mechanism is observed upon exposure to SAHA [149]. We recently showed that a new HDACi 4-(4-Chloro-2-methylphenoxy)-N-hydroxybutanamide (CMH) or droxinostat [151,152], identified using a highthroughput chemical library screen [153,154], triggered apoptosis in the breast cancer cell line MCF-7 through c-FLIPL and c-FLIPS mRNA as well as protein downregulation [151]. Interestingly, this agent induced more robust apoptosis in a doxorubicin-resistant variant of MCF-7 cells [151]. As shown in Table 1, a number of agents with modulating effects on Akt, PI3K, NF-κB, and Ras pathways, as well as an inhibitor of STAT3 have also been shown to transcriptionally silence c-FLIP expression.

#### 3.6.2. Oligonucleotide and RNAi-targeting of c-FLIP for cancer therapy

We have shown that CCRF-HSB-2 human lymphoblastic leukemia cells transfected with an antisense c-FLIP plasmid abrogated c-FLIP_S_ and c-FLIP_L_ expression and triggered a significant increase in Taxol-induced apoptosis [7]. Logan *et al.* [143] investigated whether using an antisense oligonucleotide to target c-FLIP was a clinically feasible approach (Table 2). These authors developed a novel c-FLIP-targeted antisense phosphorothioate oligonucleotide (AS PTO) and recently used it *in vitro* in transient transfection experiments and *in vivo* using xenograft models in Balb/c nude mice [143]. The AS PTO downregulated c-FLIP and resulted in caspase-8 activation and apoptosis induction in non-small cell lung cancer (NSCLC) cells, but not in normal lung cells. Similar results were observed in colorectal and prostate cancer cells. The AS PTO also sensitized cancer cells but not normal lung cells to apoptosis induced by TRAIL and increased chemotherapy-triggered apoptosis in NSCLC cells. Importantly, compared to a control non-targeted PTO, intraperitoneal delivery of c-FLIP AS PTO inhibited the growth of NSCLC xenografts and enhanced the *in vivo* antitumor effects of cisplatin. Therefore, this c-FLIP-targeted AS PTO may have potential for further pre-clinical development.

The development of RNA interference (RNAi)-based therapeutics to target the *c-FLIP* gene *in vivo* may change the way cancers are treated by inducing apoptosis [79] or by sensitizing cancers to chemotherapeutic agents. However, difficulties in siRNA design, delivery, and stability must be solved before RNAi-based therapeutics will be feasible for clinical use. We have used lipocomplexes of c-FLIP siRNA to successfully knock down the c-FLIP gene and induce spontaneous apoptosis in MCF-7 breast cancer cells *in vitro* [142] (Table 2), and *in vivo* by directly injecting the c-FLIP siRNA lipocomplexes into MCF-7 mouse xenografts [142]. Lipocomplexes of c-FLIP siRNA have also been used to successfully silence the *c-FLIP* gene and trigger spontaneous apoptosis in A549 lung cancer cells [165], HCT116 colorectal cancer cells [166], and LNCaP and PC3 prostate cancer cells [13]. Furthermore, c-FLIP siRNA lipocomplexes injected into HCT116 colorectal tumor xenografts decreased tumor growth [166]. These studies show that c-FLIP siRNA lipocomplex formulations can be used to successfully knock down the *c-FLIP* gene in various cancer cell types [76,95].

#### 3.6.3. c-FLIP degradation as a target for cancer therapy

As discussed above, c-FLIP is predominately degraded by the ubiquitin-proteasome system. Downregulation of c-FLIP_L_ and c-FLIP_S_ due to degradation is observed in cells treated with various apoptosis-inducing agents (Table 2). Cycloheximide [167] and anisomycin [153], two protein synthesis inhibitors, as well as the RNA synthesis inhibitor actinomycin D [168] have been shown to downregulate c-FLIP_L_ and c-FLIP_S_. Treating cancer cells with fluorouracil (5-FU) was also demonstrated to downregulate both isoforms in colon cancer cell lines [106,169] (Table 2).

Peroxisome proliferator-activated receptor γ (PPARγ) agonists sensitize cancer cells to TRAIL by ubiquitination and proteasome-dependent c-FLIP degradation [170-174]. Tiwary *et al.* [77] recently reported that α-tocopherol ether-linked acetic acid analogue (α-TEA) downregulation of c-FLIP is mediated by ER stress-dependent JNK/CHOP/DR5 signaling via JNK activation of Itch E3 ligase ubiquitination and involved in activation of the ER-stress-dependent events via reducing the inhibitory effect of c-FLIP on caspase-8.

Proteasome inhibitors are a new class of drugs that decrease proliferation and induce apoptosis in a variety of hematologic and solid malignancies [175-186]. Interestingly, several proteasome inhibitors lead to the downregulation of c-FLIP_L_ and c-FLIP_S_ [24,45,172]. The induction of apoptosis by the proteasome inhibitors MG-132 and PS-341 (bortezomib, Velcade^®^) in primary chronic lymphocytic leukemia (CLL) cells and the Burkitt lymphoma cell line BJAB was associated with upregulation of TRAIL and its death receptors, DR4 and DR5, and decreased c-FLIP protein expression [179]. Similarly, bortezomib decreased c-FLIP expression in multiple myeloma and human esophageal squamous cell carcinoma cell lines [180,181]. However, the effect of PS-341 on the regulation of c-FLIP expression may be cancer cell-type specific. In contrast to what was observed human esophagil cancer cell lines, Liu *et al.* reported that PS-341 upregulates DR5 as well as c-FLIP and survivin in human non-small cell lung carcinomas (NSCLC) cells [183]. As discussed earlier, c-FLIP is degraded via a ubiquitin-proteosome system. Therefore, PS-341 should increase c-FLIP and prevent apoptosis. Interestingly, Zhao *et al.* have shown that PS-341 decreases c-FLIP at the gene level [184].

The Bcr-Abl kinase inhibitor imatinib mesylate (formerly known as CGP 57148B, STI571, or Gleevec) is currently the standard therapy for chronic myeloid leukemia (CML). Hamaï *et al.* [187] reported that Imatinib mesylate increases human melanoma cell sensitivity to TRAIL-induced cell death by directly downregulating protein levels of c-FLIP variants. Interestingly, Park *et al.* [188] showed that silencing the Bcr-Abl in K562 leukemia cells led to the downregulation of c-FLIP_L_ and subsequent increase to TRAIL sensitivity.

As shown in Table 2, a number of agents known to affect various targets and signaling pathways in cancer cells also cause degradation of c-FLIP variants (189-205). Moreover, several compounds have been shown to inhibit expression of c-FLIP variants, but whether these agents cause degradation of these proteins or silence their transcription remain to be found. Nutlin-3, a small molecule antagonist of MDM2 which inhibit the p53-MDM2 interaction and activates p53 signaling was recently shown to decrease expression of c-FLIP_S_ and c-FLIP_L_ and was synergistic with TRAIL in triggering cell death [206]. Moreover, Ozarelix, a gonadotropin-releasing hormone antagonist [207], celecoxib, a cyclooxygenase-2 inhibitor [208], the chemopreventive agent, all-trans-retinyl acetate (RAc) [125], smac mimetic compounds (SMC) [180], and sunitinib, an orally administered tyrosine kinase inhibitor (TKI) [210] decreased expression of c-FLIP. Furthermore, downregulation of c-FLIP by a specific microRNAs (*i.e.*, miR-512-3p) increased taxol-induced apoptosis [211], supporting our previous report that silencing c-FLIP variants increases Taxol-triggered apoptosis [7]. Gemcitabine was also recently shown to inhibit expression of c-FLIP variant in pancreatic cancer cells [71], but whether it inhibits the transcription, enhances degradation, or prevents translation of c-FLIP remains to be found.

**Figure 4.**
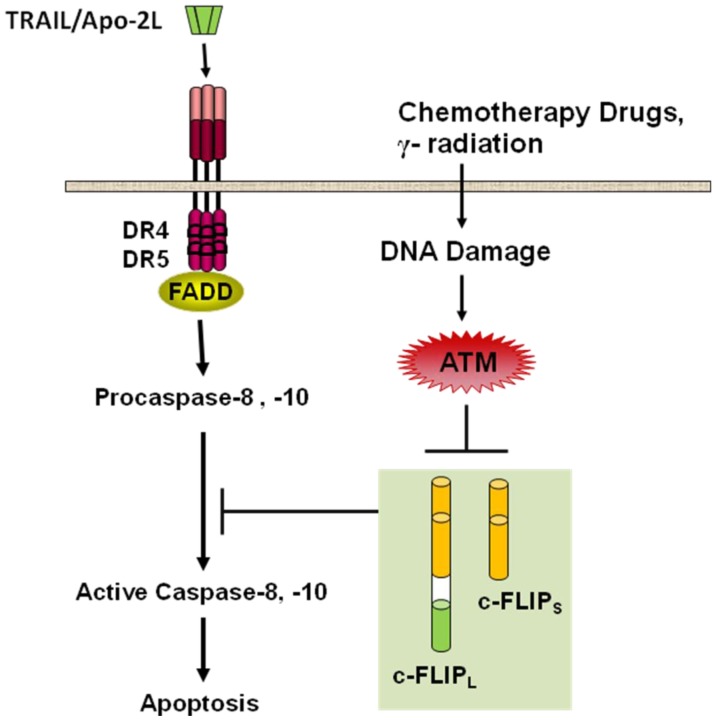
ATM kinase activity downregulates c-FLIP_L_ and c-FLIP_S_ at the protein level and connects DNA damage signaling to TRAIL-induced apoptosis signaling pathway. DNA damaging agents induce ATM activation, which promotes c-FLIP_L_ protein degradation and c-FLIP_S_ downregulation through an unknown mechanism (Stagni *et al.* [122]).

Recent data [122,212,213] clearly demonstrate that ataxia telangiectasia mutated (ATM) kinase activity modulates c-FLIP_L_ and c-FLIP_S_ protein levels in response to DNA damage (Figure 4). Moreover, the radiomimetic drug Neocarzinostatin (NCS) may trigger the down-regulation of c-FLIP isoforms by inducing the activation of the ATM kinase in response to DNA damage [122]. ATM kinase activity negatively modulates the stability of c-FLIP_L_ and c-FLIP_S_ at the protein level, thereby promoting the sensitivity to apoptosis induction by Fas (CD95/APO-1), a TRAIL-R1/R2-related death receptor [213]. NCS-triggered decrease in c-FLIP resulted in increased sensitivity to TRAIL which was inhibited by ATM kinase activity inhibition [122]. Upon NCS treatment, ATM promotes c-FLIP_L_ protein degradation through the ubiquitin-proteasome system but the mechanism of degradation of c-FLIP_S_ that ATM is linked to remains to be determined [122].

## 4. Conclusions

It is now evident that c-FLIP variants induce resistance to death receptor ligands and chemotherapeutic agents in various cancer cells and that c-FLIP may be a relevant clinical target for counteracting therapy resistant human malignancies. The current state of the art reviewed in this article suggests that targeting c-FLIP in combination with TRAIL or standard chemotherapies has therapeutic potential for treating human cancers. As discussed, various classes of agents can downregulate c-FLIP expression. However, c-FLIP has significant structural similarity to caspase-8; this makes c-FLIP a very difficult target for developing drugs that inhibit this protein directly, since small molecules capable of blocking c-FLIP's recruitment to the DISC could simultaneously inhibit the recruitment of caspase-8 and thereby inhibit apoptosis. Therefore, to reduce or inhibit c-FLIP expression, small molecules which target c-FLIP without inhibiting caspases-8 and -10 are needed. Compounds that inhibit or downregulate c-FLIP mRNA expression will particularly be of interest. As discussed above, employing a high-throughput chemical screening strategy, a small molecule inhibitor of c-FLIP, 4-(4-Chloro-2-methylphenoxy)-N-hydroxybutanamide (CMH) or droxinostat has been identified that downregulates c-FLIP_L_ and c-FLIP_S_ mRNA and protein levels, reduces cell survival, and induces apoptosis. The foregoing discussion justifies optimism that future cancer therapy will be improved by innovations that combine chemotherapy with drug resistance-reversing multi-targeted therapy, e.g., combination regimens of chemotherapy and small molecule drugs that downregulate c-FLIP.

## Figures and Tables

**Table 1.** Agents known to inhibit c-FLIP expression at the transcription level.

**Mechanism of action**	**Agent**	**References**
Generation of DNA adduct formation, intra- and interstrand crosslinks	Cisplatin, oxaliplatin	[69,70,143-146]
DNA intercalator	Doxorubicin	[147]
Topoisomerase I inhibitor	Camptothecin, 9-nitrocamptothecin (9-NC), irinotecan	[69,148]
Histone deacetylase inhibitor	Vorinostat, trichostatin, droxinostat (CMH), valproic acid, NCH-51, MS-275, romidepsin (FK228/depsipeptide), and AR-42	[149-154]
Anti-microtubule targeting agent	Lupeol (triterpene)	[155-157]
Inhibitor of IκB kinase (IKK) and NF-κB pathways	Celastrol, zerumbone, withaferin A, quinacrine	[158-160]
Inhibitor of TNFα-mediated NF-κB activation	Chrysin (flavonoid)	[161]
Inhibitor of mitogen-induced proliferative response	S-adenosylmethionine (SAMe) 5′-methylthioadenosine (MTA)	[162]
Inhibitor of signal transducer and activator of transcription 3 (STAT3)	CDDO-imidazolide (synthetic triterpenoid)	[163]
Ras/mTOR inhibitor	Salirasib	[164]

**Table 2.** Agents known to inhibit c-FLIP expression by post-transcriptional mechanisms.

**Mechanism of action**	**Agent**	**References**
Blockade of mRNA translation and RNaseH-mediated cleavage, with subsequent degradation of the mRNA:antisense DNA heteroduplex	Antisense oligonucleotide	[60]
c-FLIP RNA interference	SiRNAs	[24,45,116, 165,166]
Inhibitor of mammalian target of rapamycin (mTOR)	Rapamycin	[83]
Inhibitor of phosphorylation of (Roc) translation initiation factor 4E (eIF4E) Disrupts the eIF4E/eIF4G association	Rocaglamide	[87]
Antimicrotubule agent	Taxol (paclitaxel)	[7]
Protein synthesis inhibitors	Cyclohexamide and anisomycin	[167]
RNA synthesis inhibitor	Actinomycin D	[168,169]
Thymidylate synthase (TS) inhibitor	5-fluorouracil (5-FU)	[8,122]
PPARγ modulation	CDDO, 15-deoxy-δ (12,14)-prostaglandin J2 (15d-PGJ2), Rosiglitazone, Troglitazone	[170-174]
Proteasome inhibitors	PS-34 (bortezomib), MG-132	[80,175-186]
Bcr-Abl kinase inhibitor	Imatinib mesylate	[187,188]
Blocks the activation of NF-κB and TGF-β1/Smad signaling pathways	Silibinin (flavonoid)	[189]
Induces cyclin D1 phosphorylation, Increases ubiquitination of c-FLIP	SHetA2	[190]
Akt and NF-κB downregulation	Genistin (isoflavone)	[191]
Multikinase inhibitor	Sorafenib	[192]
Inhibitor of AKT phosphorylation	Eupatolide	[193]
Elevation of c-Jun N-terminal kinase (JNK) and its substrate c-Jun	α-TEA	[194,195]
Inhibitor of transglutaminase 2 (TG2)	Cystamine	[196]
Proteasome-mediated degradation of c-FLIP_S_	Paxilline	[197]
Inhibitor of phosphorylation of AKT (pAKT) and cellular FLICE-like inhibitory protein (c-FLIP)	Tamoxifen	[198]
Increases reactive oxygen species (ROS) and induces proteasome-dependent degradation of c-FLIP	Isoquinoline alkaloid, Berberine (BBR)	[199]
Activator of signal transduction cascades	INF-γ	[200]
Induce ROS, activator of both death receptor- and mitochondrial-mediated apoptotic pathways	β-elemene piperazine derivatives	[201]
EGFR tyrosine kinase inhibitor	AG1478	[202]
Down-regulates STAT3	Kahweol, a coffee-specific diterpene	[203]
Phosphodiesterase inhibitor	Pentoxifylline (PTX)	[204]
Phosphatidylinositol 3′-kinase inhibitor	LY294002	[205]
